# Predictors for improvement in patient-reported outcomes: post hoc analysis of a phase 3 randomized, open-label study of eculizumab and ravulizumab in complement inhibitor-naive patients with paroxysmal nocturnal hemoglobinuria

**DOI:** 10.1007/s00277-023-05483-0

**Published:** 2023-10-07

**Authors:** Hubert Schrezenmeier, Austin Kulasekararaj, Lindsay Mitchell, Régis Peffault de Latour, Timothy Devos, Shinichiro Okamoto, Richard Wells, Evan Popoff, Antoinette Cheung, Alice Wang, Ioannis Tomazos, Yogesh Patel, Jong Wook Lee

**Affiliations:** 1 Institute of Transfusion Medicine, University of Ulm, Helmholtzstraße 10, 89081 Ulm, Germany; 2 Institute for Clinical Transfusion Medicine and Immunogenetics, German Red Cross Blood Transfusion Service Baden-Württemberg-Hessen/University Hospital Ulm, Ulm, Germany; 3 King’s College Hospital, National Institute for Health and Care Research/Wellcome King’s Clinical Research Facility, London, UK; 4 Department of Hematology, University Hospital Monklands, Airdrie, UK; 5 Hôpital Saint-Louis AP-HP, Paris, France; 6 Department of Hematology, University Hospitals Leuven, Louvain, Belgium; 7 Department of Microbiology and Immunology, Laboratory of Molecular Immunology (Rega Institute), KU Leuven, Louvain, Belgium; 8 Division of Hematology, Keio University School of Medicine, Tokyo, Japan; 9 Sunnybrook Health Sciences Centre, Toronto, ON Canada; 10 Broadstreet HEOR, Vancouver, BC Canada; 11 AstraZeneca Rare Disease, Boston, MA USA; 12 Department of Hematology, College of Medicine, Seoul St. Mary’s Hospital, The Catholic University of Korea, Seoul, Republic of Korea

**Keywords:** Paroxysmal nocturnal hemoglobinuria, Quality of life, Fatigue, Eculizumab, Ravulizumab

## Abstract

Paroxysmal nocturnal hemoglobinuria (PNH) is characterized by uncontrolled terminal complement activation leading to intravascular hemolysis (IVH), thrombosis, and impairments in quality of life (QoL). The aim of this study was to identify the clinical drivers of improvement in patient-reported outcomes (PROs) in patients with PNH receiving the complement component 5 (C5) inhibitors eculizumab and ravulizumab.

This *post hoc* analysis assessed clinical outcomes and PROs from 246 complement inhibitor-naive patients with PNH enrolled in a phase 3 randomized non-inferiority study that compared the C5 inhibitors ravulizumab and eculizumab (study 301; NCT02946463). The variables of interest were lactate dehydrogenase (LDH) levels, a surrogate measure of IVH, and hemoglobin (Hb) levels. PROs were collected using Functional Assessment of Chronic Illness Therapy—Fatigue (FACIT-F) and European Organisation for Research and Treatment of Cancer, Quality of Life Questionnaire—Core 30 (EORTC QLQ-C30) to assess fatigue and QoL, respectively.

Improvements in absolute mean LDH levels were significantly associated with improvements in mean FACIT-F score (*p* = 0.0024) and EORTC QLQ-C30 global health (GH) score (*p* < 0.0001) from baseline to day 183. Improvements in scores were achieved despite a non-significant increase in Hb levels. To understand the interaction between LDH and Hb, a regression analysis was performed: LDH response with Hb improvements was a significant predictor of improvement in fatigue. The independent effect of improved Hb did not significantly affect FACIT-F or EORTC QLQ-C30 GH scores.

These findings suggest that LDH levels are an important determinant of fatigue and QoL outcomes in patients with PNH. **CTR:** NCT02946463, October 27, 2016.

**Supplementary Information:**

The online version contains supplementary material available at 10.1007/s00277-023-05483-0.

## Introduction

Paroxysmal nocturnal hemoglobinuria (PNH) is a rare, chronic, hematological disorder characterized by uncontrolled terminal complement pathway activation leading to intravascular hemolysis (IVH), as indicated by elevated lactate dehydrogenase (LDH) levels, thromboembolic events, and organ damage [1–4]. Patients with PNH commonly report symptoms of fatigue, dyspnea, and pain, which can negatively affect patients’ quality of life (QoL) [5–9]. Complement component 5 (C5) inhibitors, eculizumab and ravulizumab, are the current standards of care for treating patients with PNH [10–16].

Eculizumab is a first-generation C5 inhibitor and the first disease-specific treatment to be approved for patients with PNH, offering terminal complement inhibition through a biweekly intravenous dosing regimen [11, 12]. Ravulizumab is a second-generation C5 inhibitor, which provides the same clinical benefit as eculizumab with immediate, sustained, and complete terminal complement inhibition throughout a significantly longer intravenous dosing interval (every 8 weeks) [13–16]. The comparable efficacy and safety profiles of both treatments have been demonstrated in two phase 3, randomized, open-label, multicenter, international studies of patients with PNH who were complement inhibitor-naive and -experienced (studies 301 [NCT02946463] [17, 18] and 302 [NCT03056040] [19, 20], respectively). In both studies, ravulizumab was demonstrated to be non-inferior to eculizumab for all primary and key secondary efficacy endpoints, such as the proportion of transfusion-free patients, stabilization of hemoglobin (Hb) levels, change from baseline (ΔBL) in patient-reported outcome (PRO) measures, and normalization of LDH levels [17–20]. Patients with LDH levels of or above 1.5 × upper limit of normal (ULN) experience significantly higher risk of PNH-associated symptoms, thrombosis and death [4, 7, 8].

Improvements observed in PROs following C5 inhibitor treatment for PNH in studies 301 and 302 have been reported using the Functional Assessment of Chronic Illness Therapy—Fatigue (FACIT-F) and the European Organisation for Research and Treatment of Cancer, Quality of Life Questionnaire–Core 30 (EORTC QLQ-C30) [17–20]. These instruments have been validated to be relevant in assessing fatigue and QoL symptoms in patients with PNH [9]. The aim of this study was to identify the key clinical drivers of improvements in QoL and fatigue for patients receiving treatment for PNH. Understanding the drivers of QoL improvements is vital for developing appropriate management strategies.

Data collected from a cohort of complement inhibitor-naive patients with PNH in study 301 were used for this *post hoc* analysis, to better illustrate the treatment effect of C5 inhibitors on PROs. However, in study 302, complement inhibitor-experienced patients with PNH were clinically stable on eculizumab, and therefore changes in PRO measures were not significant and so not included in this analysis [19].

## Methods

### Study design

Details of study 301 have been described previously [17]. Briefly, this was a phase 3, multicenter, randomized, active-controlled, open-label study conducted in 123 centers in 25 countries in adult (≥ 18 years of age) complement inhibitor-naive patients with a confirmed diagnosis of PNH and high disease activity (LDH level ≥ 1.5 × ULN; 246 U/L], and one or more sign or symptom of PNH within 3 months of screening [fatigue, hemoglobinuria, abdominal pain, shortness of breath, anemia defined as Hb level < 10 g/dL or history of major adverse vascular events including thrombosis, dysphagia, erectile dysfunction, or history of packed red blood cell transfusion due to PNH]) treated with eculizumab or ravulizumab. This *post hoc* analysis utilized clinical outcomes and PROs captured from the 26-week randomized period of study 301 (i.e., up to day 183) in which patients received ravulizumab or eculizumab.

### PROs

#### FACIT-F

Fatigue was assessed at baseline and days 8, 29, 71, 127, and 183 (26 weeks) of C5 inhibitor treatment using the FACIT-F scale version 4 [21, 22]. FACIT-F is a 13-item questionnaire rated on a five-point Likert scale (0–5). The total score ranges from 0 to 52, with higher scores indicating less fatigue. Validation of the FACIT-F scale has shown it to be relevant in assessing fatigue in patients with PNH [9], with an improvement of five points considered a clinically important difference in this patient population [23].

#### EORTC QLQ-C30

QoL at baseline and days 8, 29, 71, 127, and 183 (26 weeks) of C5 inhibitor treatment was measured using the EORTC QLQ-C30 version 3.0. The EORTC QLQ-C30 contains 30 items covering: five function subscales (physical, role, emotional, cognitive, social); nine symptom subscales/items (fatigue, nausea/vomiting, pain, dyspnea, insomnia, appetite loss, constipation, diarrhea, financial difficulties); and a global health (GH) status/QoL subscale [17, 19, 24]. The current *post hoc* analysis utilized the GH subscale only. Higher GH status scores indicate better QoL (range, 0–100). Validation of the EORTC QLQ-C30 has shown it to be relevant in assessing QoL in patients with PNH [9].

### Associations between variables of interest and PROs

Variables of interest included demographic characteristics (age, sex, and body mass index), baseline and ΔBL LDH levels, baseline and ΔBL Hb levels, concomitant bone marrow disorders (aplastic anemia [AA] and myelodysplastic syndrome [MDS]), transfusion, and hematological parameters (platelet and neutrophil counts, and ratio of reticulocytes to erythrocytes [%]). Baseline LDH level was defined as the average of all available assessments before the first infusion of the study drug. For all other parameters, baseline was defined as the last available assessment before the first study drug infusion. The main laboratory variables of interest in the current analyses were Hb and LDH levels (indicators of the rate of hemolysis). Associations between mean ΔBL PRO score and laboratory variables were assessed up to 26 weeks (day 183), including absolute mean LDH level, absolute mean Hb level, mean LDH response at day 183, mean ΔBL Hb level, and mean ΔBL LDH level at day 183.

### Statistical analyses

#### Regression analysis

Regression analyses were performed using clinical and PRO data collected in study 301 to identify significant predictors for ΔBL FACIT-F score and EORTC QLQ-C30 GH score. Patient clinical and demographic characteristics were analyzed as covariates in the regression analyses. Covariates included for the full models of both PROs were baseline and ΔBL LDH levels, baseline and ΔBL Hb levels, baseline FACIT-F and EORTC QLQ-C30 GH scores, age, sex (male/female), concomitant AA or MDS (yes/no), transfusion (yes/no), treatment group (eculizumab/ravulizumab), reticulocytes/erythrocytes ratio (baseline, defined as high [≥ 2.3%], normal [0.2–2.3%], and low [≤ 0.2%]), ΔBL platelet count, ΔBL neutrophil count, and body mass index. Generalized linear regression models were fit for each outcome and set of covariates. These were conducted using the *glm* function from the *stats* package in *R* (v4.0.3; R Core Team, Austria). Interaction effects investigated included ΔBL Hb with transfusion received, ΔBL Hb among LDH responders (yes/no), ΔBL Hb in patients with AA/MDS (yes/no), transfusion received among LDH responders (yes/no), and transfusion received among patients with AA/MDS. 

#### Model selection

Starting with the full model, tests for multicollinearity were conducted with all variables included, and the model selection process proceeded iteratively by removing variables one at a time until there was no longer a positive test for multicollinearity. Variables found to be most collinear, based on individual multicollinearity tests with non-significant regression coefficients, were removed first. If only significant variables were remaining as collinear, the least significant were removed next. When there was no longer an overall positive test for multicollinearity, remaining statistically insignificant variables were removed and the fit of the model from before and after was compared.

Further details on the model selection methodology and a full list of overall and individual tests used for the multicollinearity testing can be found in Supplementary information. 

## Results

### Patient demographic and clinical characteristics

Data were available for 246 complement inhibitor-naive patients with PNH who were randomized to receive either ravulizumab (*n* = 125) or eculizumab (*n* = 121). Patient demographics and baseline clinical characteristics for these patients have previously been published [17]. Briefly, all 125 patients who received ravulizumab and 119 of the patients who received eculizumab completed the 26-week primary evaluation period. Overall, patients (mean [standard deviation; SD] age = 45.5 [15.7] years; 54.5% male) were predominantly Asian (52.4%, including Japanese race) or White (38.2%) with a mean (SD) LDH level at baseline of 1606.4 (752.7) U/L. Median (minimum, maximum) time from PNH diagnosis to treatment initiation was 3.9 (0.0, 41.0) years. In the ravulizumab group (mean [SD] age = 44.8 [15.2] years), the majority of patients were male (52.0%), Asian (57.6%), or White (34.4%) with a mean (SD) LDH level at baseline of 1633.5 (778.8) U/L. Median (minimum, maximum) time from PNH diagnosis to treatment initiation for these patients was 3.8 (0.0, 41.0) years. In the eculizumab group (mean [SD] age = 46.2 [16.2] years), patients were predominantly male (57.0%), and Asian (47.1%) or White (42.1%) with a mean (SD) LDH level at baseline of 1578.3 (727.1) U/L. Median (minimum, maximum) time from PNH diagnosis to treatment initiation in these patients was 3.9 (0.0, 34.0) years. There were no differences between ravulizumab and eculizumab treatment groups in demographics or baseline clinical characteristics [17].

### Associations between variables of interest and PROs

#### Overall results

**Fig. 1:**
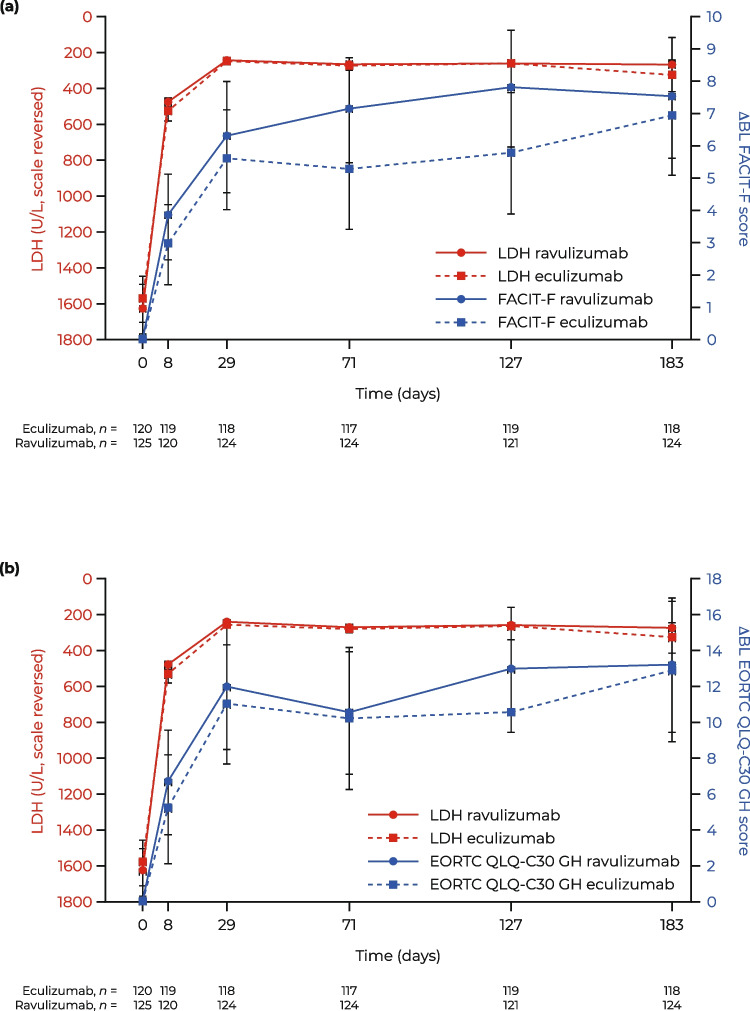

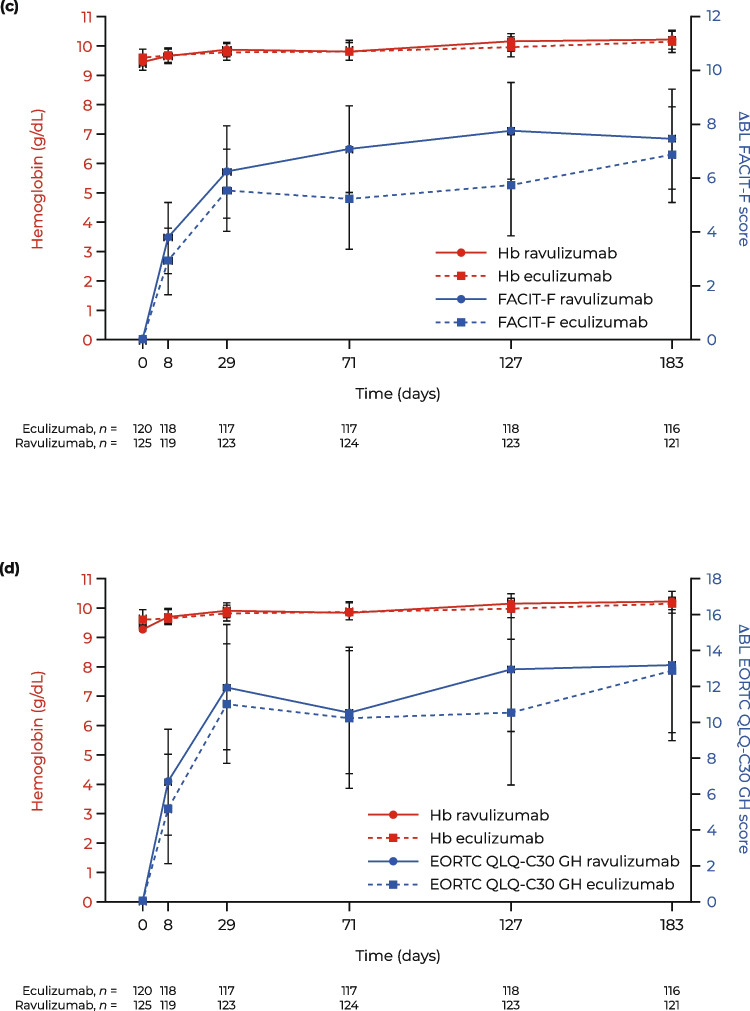
ΔBL PRO score and laboratory variables over time in patients with PNH: **A** FACIT-F score and absolute LDH level, **B** EORTC QLQ-C30 GH score and absolute LDH level, **C** FACIT-F score and absolute Hb level, and **D** EORTC QLQ-C30 GH score and absolute Hb level. Data are presented as mean (95% CI). ΔBL change from baseline, CI confidence interval, EORTC QLQ-C30 GH European Organisation for Research and Treatment of Cancer, Quality of Life Questionnaire—Core 30 global health subscale, FACIT-F Functional Assessment of Chronic Illness Therapy—Fatigue, Hb hemoglobin, LDH lactate dehydrogenase, PRO patient-reported outcome

From baseline to day 183, complement C5 inhibitor-mediated reductions in absolute mean LDH levels were significantly associated with improvements in mean FACIT-F score (*p* = 0.0024; Fig. 1a) and EORTC QLQ-C30 GH score (*p* < 0.0001; Fig. 1b). This observation was seen irrespective of treatment received (i.e., ravulizumab or eculizumab). A rapid, substantial, and sustained improvement in FACIT-F score and EORTC QLQ-C30 GH score was achieved (e.g., an increase of approximately eight points on day 28 through day 183) despite a non-significant increase in Hb levels (Fig. 1c, d; *p* = 0.4697).

#### PROs stratified by LDH response status

**Fig. 2:**
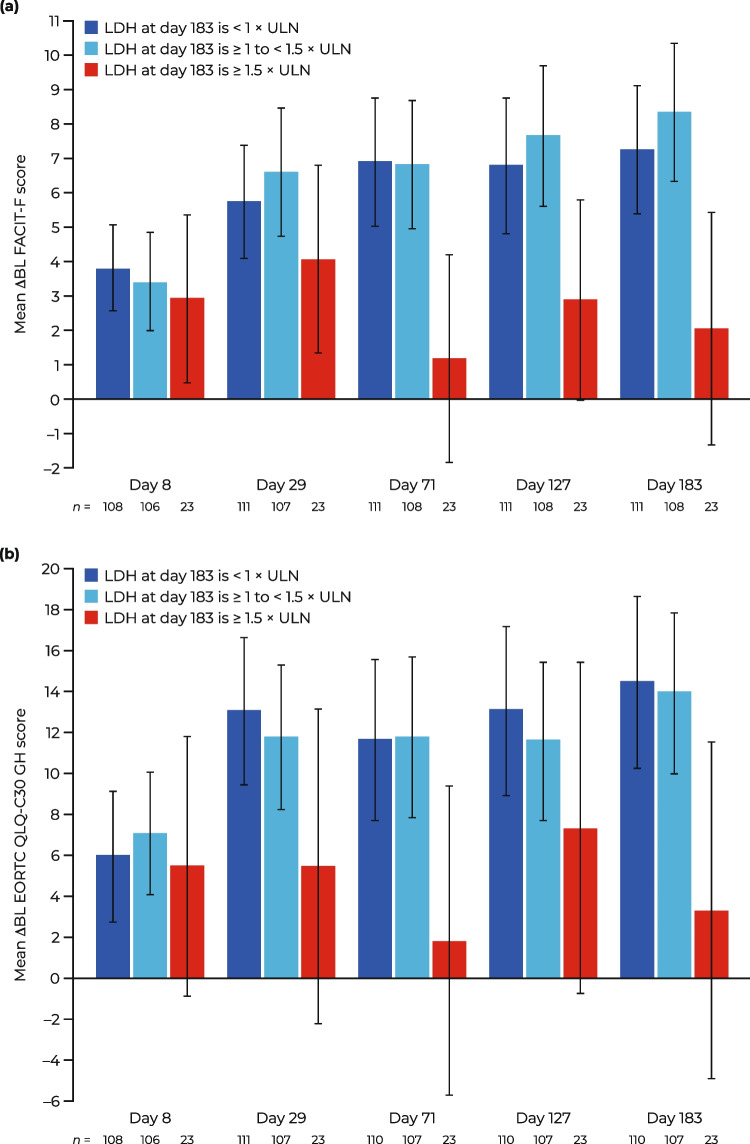
Mean ΔBL PRO scores stratified by LDH levels at day 183 in patients receiving eculizumab or ravulizumab: **A** FACIT-F and **B** EORTC QLQ-C30 GH. Data are presented as mean (95% CI). ΔBL change from baseline, CI confidence interval, EORTC QLQ-C30 GH European Organisation for Research and Treatment of Cancer, Quality of Life Questionnaire—Core 30 global health subscale, FACIT-F Functional Assessment of Chronic Illness Therapy—Fatigue, LDH lactate dehydrogenase, PRO patient-reported outcome, ULN upper limit of normal

In this study, improvement in LDH levels with C5 inhibitor treatment was associated with improvements in FACIT-F and EORTC QLQ-C30 GH scores. Compared with patients with LDH levels ≥ 1.5 × ULN at day 183 (*n* = 23; 9.5%), patients with LDH levels < 1.5 × ULN at day 183 (FACIT-F, *n* = 219; EORTC QLQ-C30 GH, *n* = 217; 90.5%, respectively) had greater overall mean improvements in ΔBL FACIT-F score and ΔBL EORTC QLQ-C30 GH score at all study time points (Fig. 2a, b). This was observed both for patients with LDH levels < 1 × ULN and LDH levels ≥ 1 to < 1.5 × ULN at day 183. However, given the small sample size in the group with LDH levels ≥ 1.5 × ULN at day 183, the differences among the three groups were marginally non-significant at a 5% confidence level. Patients with LDH levels < 1.5 × ULN at day 183 had continual improvements in mean ΔBL FACIT-F score and ΔBL EORTC QLQ-C30 GH score throughout the study period. This trend was not observed in patients with day 183 LDH levels ≥ 1.5 × ULN.

**Fig. 3:**
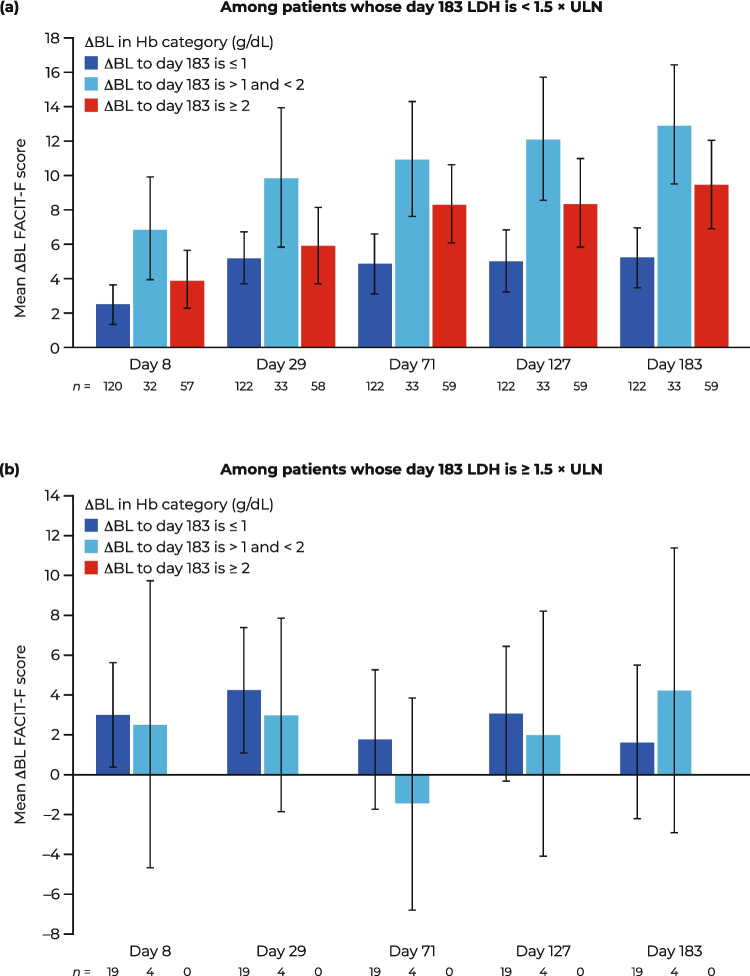
Mean ΔBL FACIT-F scores stratified by ΔBL Hb in patients receiving eculizumab or ravulizumab: **A** among patients whose LDH is < 1.5 × ULN at day 183 and **B** among patients whose LDH is ≥ 1.5 × ULN at day 183. Data are presented as mean (95% CI). ΔBL change from baseline, CI confidence interval, FACIT-F Functional Assessment of Chronic Illness Therapy—Fatigue, Hb hemoglobin, LDH lactate dehydrogenase, ULN upper limit of normal

In patients with LDH levels < 1.5 × ULN at day 183, improvements in mean Hb level were associated with continual improvements in mean FACIT-F score throughout the study period (Fig. 3a). This was observed in those with ΔBL and increase in Hb levels of ≥ 1 g/dL at day 183. Patients who had the greatest mean ΔBL Hb level at day 183 (i.e., mean ΔBL Hb level ≥ 2 g/dL) did not demonstrate the greatest improvements in FACIT-F score. There were no clear trends in ΔBL FACIT-F score with ΔBL Hb level at day 183 in patients who did not have LDH levels < 1.5 × ULN at day 183 (Fig. 3b).

### Regression analysis

Significant predictors of improvement in FACIT-F score (*p* < 0.05) included clinical and demographic characteristics as well as treatment-related outcomes. These were: baseline FACIT-F score, younger age, male gender, reduced reticulocytes/erythrocytes ratio, reductions in LDH levels from baseline to day 183, LDH response (LDH ≤ 1.5 × ULN) at day 183 with improvements in Hb levels from baseline, and transfusion requirement with no LDH response at day 183 (Table 1).

**Table 1:** Predictors of ΔBL FACIT-F and EORTC QLQ-C30 GH at day 183 in patients receiving eculizumab or ravulizumab

Term	Estimate	SE	*p* value^a^
FACIT-F, *R*^2^ = 0.60			
Intercept	38.6986	2.7605	0.0000
ΔBL LDH	− 0.0019	0.0006	**0.0024**
Baseline FACIT-F score	− 0.7264	0.0458	**0.0000**
Age	− 0.0625	0.0295	**0.0350**
Sex (female)	− 1.9503	0.9265	**0.0364**
High reticulocytes/erythrocytes ratio	− 4.1013	1.7214	**0.0181**
No LDH response^b^ at day 183 with ΔBL Hb	− 0.1292	0.1302	0.3220
LDH response at day 183 with ΔBL Hb	0.0580	0.0263	**0.0285**
Received transfusion with no LDH response^b^ at day 183	− 4.9443	2.0253	**0.0154**
Received transfusion with LDH response^b^ at day 183	− 0.3958	1.2255	0.7471
EORTC QLQ-C30 GH, *R*^2^ = 0.64			
Intercept	47.2109	5.1292	0.0000
ΔBL LDH	− 0.0064	0.0014	**0.0000**
Baseline EORTC QLQ-C30 GH score	− 0.6379	0.0533	**0.0000**
High reticulocytes/erythrocytes ratio	− 8.1078	4.1182	0.0502
ΔBL Hb at day 183 and did not receive transfusion	0.1090	0.0691	0.1159
ΔBL Hb at day 183 and received transfusion	0.2507	0.1070	**0.0200**

*ΔBL* change from baseline, *EORTC QLQ-C30 GH* European Organisation for Research and Treatment of Cancer Quality of Life Questionnaire—Core 30 global health subscale, *FACIT-F* Functional Assessment of Chronic Illness Therapy—Fatigue, *Hb* hemoglobin, *LDH* lactate dehydrogenase, *SE* standard error, *ULN* upper limit of normal

^a^Bolding indicates statistical significance (*p* < 0.05)

^b^Response defined as LDH level ≤ 1.5 × ULN

Low baseline EORTC QLQ-C30 GH score, reductions in LDH levels from baseline to day 183, and improvement in Hb levels from baseline to day 183 with transfusion were significant predictors of EORTC QLQ-C30 GH score improvement (*p* < 0.05). The independent effect of Hb levels, whether as an improvement from baseline to day 183 or value at baseline, was not a statistically significant predictor of improvement in either FACIT-F or EORTC QLQ-C30 GH score at day 183 and was therefore not included in the final model.

## Discussion

This *post hoc* analysis aimed to further understand the interactions between variables associated with improvements in PNH and PROs in patients treated with eculizumab or ravulizumab, who were previously naive to complement inhibitor therapy. Variables assessed included LDH, the increased levels of which are associated with more severe disease [4, 7, 8].

Individuals with PNH frequently experience symptoms of fatigue, which contribute to impaired QoL [5, 25]. EORTC QLQ-C-30 and FACIT-F have been validated to be relevant in assessing QoL and fatigue symptoms, respectively, in patients with PNH [9]. In addition, several large trials in PNH also used EORTC QLQ-C-30 (TRIUMPH [NCT00122330] [26], SHEPHERD [NCT00130000] [27]). More recently, disease-specific tools for assessing QoL and PROs in PNH have been developed including the Quality of Life Questionnaire for AA and PNH (QLQ-AA/PNH) and the PRO questionnaire for AA and PNH (PRO-AA/PNH) [28, 29]. The PNH Symptom Questionnaire (PNH-SQ) has been developed to assess daily PNH symptoms of patients [30]. However, further validation of PRO-AA/PNH and determination of the psychometric properties of QLQ-AA/PNH and PNH-SQ are required [28–30].

PNH is characterized by a deficiency of cell surface glycosylphosphatidylinositol-anchored proteins, including the complement regulatory proteins CD55 and CD59. The absence of CD55 and CD59 causes uncontrolled terminal complement activity that leads to IVH, which is indicated by increased LDH levels. IVH releases large amounts of free Hb causing nitric oxide depletion which manifests clinically as symptoms of fatigue, dysphagia, abdominal pain, and erectile dysfunction [31–34]. Terminal complement activity may also contribute to fatigue through the release of inflammatory cytokines such as interleukin (IL)-6, IL-8, and tumor necrosis factor-α [35]. This may explain why improvements in fatigue have been observed without complete resolution of anemia [26]. The results of this *post hoc* analysis suggest that a reduction in LDH levels, a biomarker of hemolysis, was a strong predictor of improvements in both fatigue, assessed by FACIT-F, and QoL, assessed by EORTC QLQ-C-30. Other predictors of improvement in FACIT-F included a lower baseline FACIT-F score (i.e., worse fatigue), younger age, male gender, reduced reticulocytes/erythrocytes ratio, LDH response with improvements in Hb levels, and transfusion without LDH response. ΔBL Hb without LDH response at day 183 and transfusion with LDH response at day 183 were not significant predictors of improvement in FACIT-F. This observation of interaction effects in the regression analysis aligns with the notion that improvement of anemia alone is not a strong predictor of improvement of QoL. Other predictors of improvement in QoL included a low baseline QoL score and improvement in Hb levels with transfusion. High reticulocytes/erythrocytes ratio and ΔBL Hb without transfusion were not significant predictors of improvement in QoL.

In study 301, C5 inhibition with eculizumab or ravulizumab demonstrated significant improvements in key clinical parameters associated with PNH, with 219 patients (90.5%) achieving LDH < 1.5 × ULN at day 183. The improvement seen in LDH levels was associated with improvements in patient fatigue and QoL, as assessed by FACIT-F and EORTC QLQ-C30 GH, respectively. In other studies, improvements in Hb levels associated with C5 inhibition have previously been reported to be associated with QoL outcomes in patients with PNH [36]. The phase 3 open-label PEGASUS trial, which compared the efficacy and safety of pegcetacoplan with eculizumab for the treatment of PNH in patients already receiving eculizumab, reported ΔBL Hb level to week 16 as the primary endpoint [37]. In that trial, the authors reported significant improvements in Hb level which translated to improvements in transfusion dependence and fatigue. This is in contrast to study 301, in which patients with the greatest mean ΔBL Hb level (≥ 2 g/dL) following C5 inhibitor treatment did not demonstrate the greatest improvements in FACIT-F scores. However, there are key population and methodological differences between the two studies, which may influence these findings. Importantly, patients in the PEGASUS trial had prior complement inhibitor treatment and Hb levels < 10.5 g/dL at screening. In addition, the PEGASUS trial did not directly assess the ability of Hb, LDH, and other variables to predict improvement in fatigue and QoL and the EORTC QLQ-C30 score was not used to measure QoL, making it difficult to draw comparisons between the results of both studies.

To understand the interaction between LDH and Hb in study 301, a regression analysis was performed. The interaction of LDH response with improvements in Hb level was a significant predictor of improvement in fatigue in the current study, demonstrating the significance of LDH level in driving PROs. This is similar to a previous multivariate analysis of data from TRIUMPH, a phase 3 trial of eculizumab and placebo, which demonstrated that a reduction in IVH was better than improvements in anemia in predicting improvements in fatigue [26, 38, 39]. Improved Hb level did not independently affect FACIT-F or EORTC QLQ-C30 GH scores in the current analysis. A rapid improvement in PROs during the first 28 days of treatment with C5 inhibitors was observed despite non-significant increases in Hb levels. However, the combined effects of improved Hb level with reduction in blood transfusion and improved Hb level with reduction in LDH to < 1.5 × ULN were significant predictors for improvement in EORTC QLQ-C30 GH and FACIT-F scores, respectively. These results illustrate the significance of decreased LDH levels in driving QoL and fatigue outcomes in patients with PNH, highlighting the detrimental effect of IVH and the importance of maintaining LDH levels below 1.5 × ULN for disease control and QoL.

There are limitations to this study that must be considered. Although study 301 collected data covering both physical functioning and fatigue subscales of the EORTC QLQ-C30, only the GH subscale was used for this analysis. Future research may benefit from incorporating additional domains to describe the interactions between clinical outcomes and PROs further. The use of PNH-specific PRO and QoL tools may also be considered in future studies once further validation and psychometric properties have been established. The changes in LDH levels linked to improvements in fatigue and QoL beyond 26 weeks remain unknown. Additionally, study 301 was a controlled trial and may not be reflective of real-world clinical practice.

## Conclusions

Understanding key clinical drivers of improvements in QoL and fatigue during C5 inhibitor therapy is important for developing appropriate management strategies for patients with PNH. In conclusion, these findings suggest that LDH level, as a surrogate parameter for IVH, is a key determinant of fatigue and QoL outcomes in patients with PNH. Treatment goals, particularly for C5-inhibitor naive patients, should focus on the improvement of LDH levels and subsequent avoidance of IVH, which C5 inhibitors have been demonstrated to successfully achieve.

### Supplementary Information

Below is the link to the electronic supplementary material.

Supplementary file1 (DOCX 49 KB)

## Data Availability

Alexion, AstraZeneca Rare Disease will consider requests for disclosure of clinical study participant-level data provided that participant privacy is assured through methods such as data de-identification, pseudonymization, or anonymization (as required by applicable law), and if such disclosure was included in the relevant study informed-consent form or similar documentation. Qualified academic investigators may request participant-level clinical data and supporting documents (statistical analysis plan and protocol) pertaining to Alexion-sponsored studies. Further details regarding data availability and instructions for requesting information are available in the Alexion Clinical Trials Disclosure and Transparency Policy at http://alexion.com/our-research/research-and-development. Link to data-request form: https://alexion.com/contact-alexion/medical-information.
