# Supplementary material for: Predictors for improvement in patient-reported outcomes: post hoc analysis of a phase 3 randomized, open-label study of eculizumab and ravulizumab in complement inhibitor-naive patients with paroxysmal nocturnal hemoglobinuria
Source: Ann Hematol. 2023 Oct 7;103(1):5–15. doi: 10.1007/s00277-023-05483-0 (PMC10761522; doi:10.1007/s00277-023-05483-0)
Supplement: Supplementary file 1 — Supplementary file1 (DOCX 49 KB) [file 277_2023_5483_MOESM1_ESM.docx]

Predictors for improvement in patient-reported outcomes: post hoc analysis of a phase 3 randomized, open-label study of eculizumab and ravulizumab in complement inhibitor-naive patients with paroxysmal nocturnal hemoglobinuria

*Annals of Hematology*

Hubert Schrezenmeier^1,2^, Austin Kulasekararaj^3^, Lindsay Mitchell^4^, Régis Peffault de Latour^5^, Timothy Devos^6,7^, Shinichiro Okamoto^8^, Richard Wells^9^, Evan Popoff^10^, Antoinette Cheung^10^, Alice Wang^11^, Ioannis Tomazos^11*^, Yogesh Patel^11^, Jong Wook Lee^12^

^1^Institute of Transfusion Medicine, University of Ulm, Ulm, Germany
^2^Institute for Clinical Transfusion Medicine and Immunogenetics, German Red Cross Blood Transfusion Service Baden-Württemberg-Hessen/University Hospital Ulm, Ulm, Germany ^3^King’s College Hospital, National Institute for Health and Care Research/Wellcome King’s Clinical Research Facility, London, UK
^4^Department of Hematology, University Hospital Monklands, Airdrie, UK
^5^Hôpital Saint-Louis AP-HP, Paris, France
^6^Department of Hematology, University Hospitals Leuven, Leuven, Belgium
^7^Department of Microbiology and Immunology, Laboratory of Molecular Immunology (Rega Institute), KU Leuven, Leuven, Belgium
^8^Division of Hematology, Keio University School of Medicine, Tokyo, Japan
^9^Sunnybrook Health Sciences Centre, Toronto, ON, Canada
^10^Broadstreet HEOR, Vancouver, BC, Canada
^11^Alexion, AstraZeneca Rare Disease, Boston, MA, USA
^12^Department of Hematology, Seoul St. Mary’s Hospital, College of Medicine, The Catholic University of Korea, Seoul, Republic of Korea

*Affiliation at time of study

**Corresponding author:** Professor Hubert Schrezenmeier

Email: [h.schrezenmeier@blutspende.de](mailto:h.schrezenmeier@blutspende.de)

# Supplementary Information

## Tests for multicollinearity

All tests contained within the mctest (v1.3.1) package in *R* (v4.0.3) were conducted. If any of the six overall tests flagged a positive result, the model selection process continued. For individual tests, we focused on the commonly used variance inflation factor (VIF) and selected the auxiliary F-test (Fi) to represent the remaining tests because their relative magnitudes were consistent. The VIF is computed by regressing each predictor (*i*) against each other predictor. This gives an R-squared value ($R_{i}^{2}$).

VIF = $\frac{1}{1 - R_{i}^{2}}$

For the VIF, a general rule of thumb is that scores over 5 are moderately multicollinear and scores over 10 are very problematic. A high VIF on its own does not necessarily indicate a problem with that specific variable. It could indicate that that variable explains a lot on its own, and that we do not need the other variables in the model that it is collinear with.

Overall tests for multicollinearity:

- Determinant of the correlation matrix [1]
- Farrar test of chi-square for presence of multicollinearity [2]
- Red indicator [3]
- Sum of lambda inverse values [4]
- Theil's indicator [5]
- Condition number [6]

Individual tests for multicollinearity:

- VIF [7]
- Tolerance (TOL) score = 1/(VIF)
- Farrar F-test (Wi) for determination of multicollinearity [2]
- Fi for relationship between F and R-square [8]
- Leamer's method [9]
- Corrected VIF (CVIF) [10]
- Klein's rule [11]
- IND1 and IND2 [12]

### References

1. Cooley VW, Lohnes P (1971) Multivariate data analysis

2. Farrar D, Glauber R (1967) Multicollinearity in regression analysis: the problem revisited

3. Kovács P, Petres T, Tóth L (2005) A new measure of multicollinearity in linear regression models. Int Stat Rev 73:405–12.

4. Chatterjee S, Hadi AS (1977) Regression Analysis by Example. 4th ed. New York

5. Theil H (1971) Principles of Econometrics. New York

6. Belsley DA, Kuh E, Welsch RW (1980) Regression Diagnostics: Identifying Influential Data and Sources of Collinearity. New York

7. Marquaridt DW (1970) Generalized inverses, ridge regression, biased linear estimation, and nonlinear estimation. Technometrics 12:591–612. <https://doi.org/10.1080/00401706.1970.10488699>

8. Gujarati D, Porter C (2008) Basic Econometrics (5th ed.). New York

9. Greene WH (2000) Econometric analysis (4th ed.). Upper Saddle River, N.J.

10. Curto JD, Pinto JC (2011) The corrected VIF (CVIF). J Appl Stat 38:1499–507. <https://doi.org/10.1080/02664763.2010.505956>

11. Klein LR. (1962) An Introduction to Econometrics (2nd ed.). New Jersey

12. Imdad Ullah M, Altaf S, Ahmed M (2019) Some new diagnostics of multicollinearity in linear regression model. Sains Malaysiana 48:2051–60. <https://doi.org/10.17576/jsm-2019-4809-26>
